# Analyzing Modern Biomolecules: The Revolution of Nucleic-Acid Sequencing – Review

**DOI:** 10.3390/biom11081111

**Published:** 2021-07-28

**Authors:** Gabriel Dorado, Sergio Gálvez, Teresa E. Rosales, Víctor F. Vásquez, Pilar Hernández

**Affiliations:** 1 Dep. Bioquímica y Biología Molecular, Campus Rabanales C6-1-E17, Campus de Excelencia Internacional Agroalimentario (ceiA3), Universidad de Córdoba, 14071 Córdoba, Spain; 2 Dep. Lenguajes y Ciencias de la Computación, Boulevard Louis Pasteur 35, Universidad de Málaga, 29071 Málaga, Spain; galvez@uma.es; 3 Laboratorio de Arqueobiología, Avda. Universitaria s/n, Universidad Nacional de Trujillo, 13011 Trujillo, Peru; teresa1905@hotmail.com; 4 Centro de Investigaciones Arqueobiológicas y Paleoecológicas Andinas Arqueobios, Martínez de Companón 430-Bajo 100, Urbanización San Andres, 13088 Trujillo, Peru; vivasa2401@yahoo.com; 5 Instituto de Agricultura Sostenible (IAS), Consejo Superior de Investigaciones Científicas (CSIC), Alameda del Obispo s/n, 14080 Córdoba, Spain; phernandez@ias.csic.es

**Keywords:** first-generation sequencing (FGS), second-generation sequencing (SGS), third-generation sequencing (TGS), high-throughput sequencing (HTS), next-generation sequencing (NGS), structural genomics, functional genomics, epigenomics, metagenomics

## Abstract

Recent developments have revolutionized the study of biomolecules. Among them are molecular markers, amplification and sequencing of nucleic acids. The latter is classified into three generations. The first allows to sequence small DNA fragments. The second one increases throughput, reducing turnaround and pricing, and is therefore more convenient to sequence full genomes and transcriptomes. The third generation is currently pushing technology to its limits, being able to sequence single molecules, without previous amplification, which was previously impossible. Besides, this represents a new revolution, allowing researchers to directly sequence RNA without previous retrotranscription. These technologies are having a significant impact on different areas, such as medicine, agronomy, ecology and biotechnology. Additionally, the study of biomolecules is revealing interesting evolutionary information. That includes deciphering what makes us human, including phenomena like non-coding RNA expansion. All this is redefining the concept of gene and transcript. Basic analyses and applications are now facilitated with new genome editing tools, such as CRISPR. All these developments, in general, and nucleic-acid sequencing, in particular, are opening a new exciting era of biomolecule analyses and applications, including personalized medicine, and diagnosis and prevention of diseases for humans and other animals.

## 1. Three Sequencing Generations

**Figure 1:**
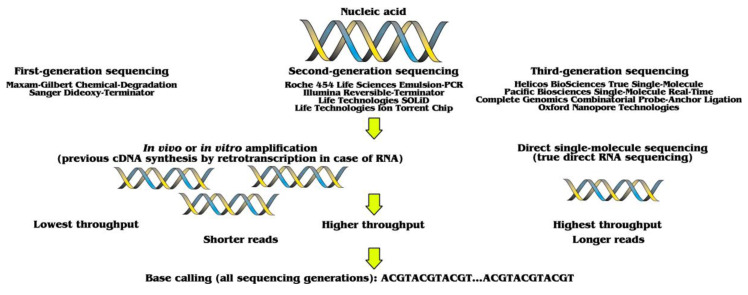
**Comparison of sequencing platforms.** FGS allows to sequence small fragments of DNA. SGS represents a significant increase in throughput. Finally, besides generating much longer reads, TGS can sequence single molecules without previous RNA retrotranscription or DNA amplification. Such a breakthrough allows to directly sequence RNA.

Analyses of biomolecules have been revolutionized by different technologies, including: (i) molecular-marker design; (ii) amplification of deoxyribonucleic acids (DNA); and (iii) nucleic-acid sequencing. The latter allows to read the code of life, being initially developed for DNA. That also allows to indirectly sequence ribonucleic acids (RNA), after retrotranscription into complementary DNA (cDNA). This is known by the misleading name of RNA sequencing (RNA-seq), instead of the more appropriate cDNA sequencing (cDNA-seq) terminology. Actually, it is not a true sequencing of native RNA, but of cDNA instead, with all biases that might be associated with such a process. Initially, all this required the previous amplification of DNA or cDNA by in vivo molecular cloning into suitable hosts, like *Escherichia coli*. Such processes typically required several years of dedicated work. The methodology was significantly enhanced by in vitro amplification technologies, such as polymerase chain reaction (PCR). A significant step forward was accomplished with the development of platforms capable of massive parallel sequencing, as well as sequencing single molecules of nucleic acids. That way, it is now possible to directly sequence not only DNA, without previous amplification or labeling steps, but also RNA, without previous retrotranscription. Nucleic-acid sequencing technologies are classified as first-generation sequencing (FGS), second-generation sequencing (SGS) and third-generation sequencing (TGS). High-throughput sequencing (HTS) methodologies, such as SGS and TGS, are sometimes known with the ambiguous “next”-generation sequencing (NGS) terminology. Such platforms are briefly described below (Figure 1).

FGS platforms include (i) chemical degradation (CD; Maxam-Gilbert); and (ii) dideoxy terminator (ddT; Sanger). They can sequence short fragments of DNA. FGS methods were revolutionary when developed, since they allowed researchers to sequence DNA for the first time. Sanger’s approach was further optimized (e.g., using fluorescent labels, instead of the original radioactive ones). In vitro amplification replaced tedious and time-consuming molecular cloning protocols, drastically reducing workflow times from several years to just months or minutes. Thus, it became very popular, being extensively used for decades to sequence short stretches of DNA. However, FGS approaches are expensive, time consuming and with low throughput. Therefore, they are not practical to sequence full genomes or transcriptomes. Indeed, the Human Genome Project using such a platform took 15 years, at a cost of three million milliard USD, even after optimizations that increased reading lengths and reduced errors, allowing researchers to finish it in half the time than previously expected at the time [1]. Bioinformatics tools were used to generate contigs, scaffolds, chromosome assemblies and full genome annotation, for such de novo sequencing. A large number of reactions and sequencing machines were used, as well as an intense labor force.

Subsequently, SGS of DNA represented a new breakthrough in biomolecule research, allowing to sequence genomes at an affordable time–cost scale. Indeed, SGS overcomes some limitations of FGS, using different approaches, corresponding to different commercial platforms, including: (i) emulsion PCR (emPCR; Roche-454 Life Sciences; Basel, Switzerland); (ii) reversible-terminator (RT; Illumina; San Diego, CA, USA); (iii) sequencing by oligonucleotide ligation and detection (SOLiD; Thermo Fisher Scientific-Life Technologies; Waltham, MA, USA); and (iv) ion torrent (IonT) chip, from the same manufacturer. Yet, albeit revolutionary in relation to FGS, SGS still has some shortcomings. They include the requirements to amplify DNA or retrotranscribe RNA. Indeed, that may introduce sequence biases, due to DNA polymerase or retrotranscriptase errors (generating mutations), with subsequent errors in the sequence readings [2]. Failure to properly read sequences may also arise in repetitive stretches (including homopolymers) and CG-rich regions, due to enzymatic limitations of DNA polymerases. Besides, the typical short-readings of SGS may pose insurmountable hindrances, since they may be difficult, if not impossible, to be accurately assembled, mainly in the absence of a reference genome. The rationale is that similar or identical short fragments may be located at different genome sites. So, it may become impossible to map a particular short sequence to any specific site, amongst the multiple potential targets available in the genome [3]. Also, as with FGS, SGS can be applied to sequence DNA, but cannot directly sequence RNA molecules.

Fortunately, TGS of nucleic acids represents a new revolution [4]. Its key advantages stem from the fact that it can directly sequence long single nucleic-acid molecules. Thus, it allows true and direct RNA-sequencing (DRS) and direct DNA-sequencing (DDS) of molecules, without previous retrotranscription or amplification, respectively. Therefore, it prevents biases associated with such steps [2]. Several TGS platforms have been released, including: (i) true single-molecule sequencing (tSMS; Helicos BioSciences; Cambridge, MA, USA); (ii) single-molecule real-time (SMRT; Pacific Biosciences; PacBio; Menlo Park, CA, USA); (iii) combinatorial probe-anchor ligation (cPAL; BGI Group-Complete Genomics; Shenzhen, China); and (iv) nanopore (NP) sequencing (Oxford Nanopore Technologies; Oxford, UK). The approaches from Helicos and Oxford allow direct sequencing of DNA or RNA. Additionally, long-read sequencing platforms have great potential in many research areas [5,6], allowing annotations without, or with lower, assembly requirements (depending on the source sequence length), streamlining data processing workflows [7]. In particular, Pacific Biosciences generates long reads of 20 kb on average, reaching 300 kb [8]. Nanopore sequencing can generate 30 kb reads, reaching even 2.3 Mb [9]. However, some shortcomings of TGS (like the requirement for higher nucleic acid concentrations and higher error rates than other platforms) should be properly addressed, to reach its full potential [4,9,10,11,12].

## 2. Applications of Nucleic-Acid Sequencing

Optimizations in experimental protocols and improvement of commercial sequencing platforms have allowed an exponential growth of applications of nucleic-acid sequencing. Indeed, there is currently a new revolution, as shown by the exponential growth of publications, regarding the possibility to sequence DNA and RNA from since-cells, as well as single organelles (mitochondria and chloroplasts) [13]. Special emphasis is now focused on integrating different -omics technologies, such as genomics (usually, DNA), transcriptomics (RNA), proteomics (peptides, like proteins), epigenomics (epigenetic factors) and metabolomics (metabolites), that eventually influence phenotypes in health and disease [14,15,16,17]. Furthermore, a combination of multi-omics techniques, complemented with morphological and physiological ones, allows a holistic approach to deciphering biological systems [18,19].

The huge amount of data generated, mainly by SGS and TGS, is demanding new software and hardware developments. Thus, mathematical tools, including statistical and bioinformatics ones involving artificial intelligence (AI), machine learning (ML) and dedicated neural network hardware (like neural engines), are being developed to better analyze the big data generated [20,21]. Some bioinformatics tools have been designed to reduce sequencing errors, like the in vivo genome diversity analyzer (iGDA), which can identify low frequency (down to 0.2%) single-nucleotide polymorphisms (SNP) [12]. Besides, recent developments are allowing to enrich nucleic-acids from samples using genome-editing tools, like clustered regularly-interspaced short palindromic repeats (CRISPR) [22]. A recent example of the relevance of new nucleic-acid sequencing technologies can be illustrated with their use to fight the current pandemic of coronavirus disease 2019 (COVID-19), caused by severe acute respiratory syndrome coronavirus 2 (SARS-CoV-2) [23].

Interestingly, the National Aeronautics and Space Administration (NASA) <https://www.nasa.gov> (accessed on 27 July 2021) has recently tested the MinION Mk1B portable sequencer (handheld; dimensions of 10′5 × 3′3 × 2′3 cm and just 87 g of weight) from Oxford Nanopore Technologies <https://nanoporetech.com/products/minion> (accessed on 27 July 2021) for astrobiology [24,25,26,27]. It can sequence nucleic acids in just 10 min, at an affordable price of just 1000 USD for the starter kit (including MinION and all materials for two runs). Traditionally, crew members of the International Space Station (ISS) have been routinely monitored for health status, including DNA tests. This requires sending samples to planet Earth for analyses. Since the MinION works in microgravity, it allows the identification of biological entities and the diagnosis of diseases in space. It could be also used in future missions to Mars or other places, allowing to search for and identify nucleic-acid-based life on such places [24]. Of course, these are uncertain astrobiology projects. Indeed, if it exists, finding life outside our planet is not an easy task. Time will tell, but such a miniature sequencer also has interesting applications on Earth, including in situ ecological studies. Some significant applications of nucleic-acid sequencing are described below.

### 2.1. Structural Genomics

Nucleic-acid sequencing allows the identification of specific nucleotide sequences of biological entities. That is interesting per se, as well as to compare mutations (polymorphisms) between molecules (genotyping). There is a plethora of applications of structural genomics, including, among others: (i) comparative genomics, to discover identities and differences between molecules; (ii) chromatin profiling, to identify regulatory regions; (iii) diagnostic and treatment of diseases, with great potential for agronomy, pharmacology and medicine; (iv) marker-assisted breeding, significantly accelerating selection; (v) certification of protected designations of origin (PDO), protected geographical indication (PGI) and traditional specialties guaranteed (TSG) for foodstuffs; (vi) identification of contaminations and frauds in foodstuffs; (vii) illegal traffic monitoring, e.g., protected species and their remains; (viii) biodiversity and ecological research, including management of germplasm banks; (ix) linking genotypes to phenotypes, including behavior; (x) bioengineering, with great impact on agronomy, medicine and biotechnology; (xi) origin of life studies; and (xii) synthetic biology, further allowing the investigation of the origin of life, and also with significant biotechnological potential. Nucleic-acid sequencing is relevant when studying any biological entity or its parts, virtually covering all life-science-related areas. To illustrate such applications, some examples of this revolution in biomolecule analyses are described below, with emphasis on the most recent ones, mostly related to medical applications.

As an example of the relevance of structural genomics, the Human Genome Project opened the door for whole-genome resequencing and targeted applications, such as exome resequencing. This has important implications in disease diagnostics and clinical treatments. Its full potential is being currently expanded with SGS and TGS platforms. This should allow further accomplishments, with the promise of 100 USD human genome resequencing. Genotyping is traditionally carried out using molecular markers or sequencing specific targeted common/known loci. Whole-genome sequencing (WGS) represents the ultimate molecular marker, allowing such genetic profiling with an unprecedented power. This includes different biotechnological areas, such as pharmacogenetic profiling [28]. Indeed, twins and even two cells from the same organism can now be differentiated with such a powerful tool. In this manner, new sequencing technologies are allowing researchers to better diagnose and analyze diseases [29]. Amongst the many examples available are the fight against complex diseases such as cancer [13,30] and neuromuscular disorders (NMD), involving more than 600 genes, affecting one in every thousand persons worldwide [31], and structural variations (SV), as shown for conditions such as autism. Interestingly, some of them are related to non-coding sequences [32].

Besides nuclear DNA in eukaryotes, organelle genomes should also be considered. For instance, they are relevant when analyzing mitochondrial disorders. New sequencing platforms have revolutionized diagnostics of such diseases, mainly exome and whole-genome approaches, including mitochondrial heteroplasmy [33]. Nevertheless, a holistic -omics approach is needed to generate more comprehensive results, also requiring new bioinformatics tools to properly analyze them [34,35,36,37,38].

New sequencing technologies are also allowing to study beneficial and pathogenic biological entities, representing significant advances for medical diagnosis and therapy [39], as well as agronomy [40,41], allowing researchers to sequence even single cells [42]. Horizontal gene transfer (HGT) in microbial communities is also important. This can generate antibiotic resistance, with significant relevance in different research areas [43]. Additionally, another of the most interesting applications of genome sequencing is personalized medicine, like sequencing single gametes [44,45]. Nucleic acids can also be used to store any kind of information in a compact and efficient way which can be retrieved by sequencing and decoding [46].

### 2.2. Functional Genomics

Transcriptomics was initially addressed retrotranscribing RNA into cDNA and further in vivo molecular cloning. That allowed the sequencing of specific molecules using FGS. The procedure was significantly optimized with in vitro amplification methodologies, such as PCR. Furthermore, SGS opened the door to sequencing full transcriptomes at an affordable cost, which was another revolution in biomolecule research. However, the most significant breakthrough came from TGS, since it allowed the direct sequencing of RNA, without retrotranscription or amplification steps, avoiding the biases related to them. Like structural genomics described above, functional genomics or transcriptomics are used in different fields, such as agronomy and medicine. Abiotic and biotic stresses, as well as disease tolerance and resistance, can be analyzed in plants and animals at the molecular level, with significant implications in breeding programs and health [47]. Such strategies can be coupled with ML to optimize big data analyses [48,49]. Genomics-assisted breeding (GAB) allows to improve the germplasm [50]. Besides, multiple stress combinations can be studied [51]. Systems biology strategies are particularly interesting, implementing holistic approaches in these scenarios, integrating different -omics and bioinformatics tools [52]. This is especially relevant in the current trend of global warming and climate change [53,54,55,56,57]. As with structural genomics, studies of functional genomics are growing at an exponential rate in different areas related to biological entities. Some relevant examples are described below, with emphasis on medical applications.

New sequencing platforms, in general, and TGS, in particular, with longer reads of full-length transcripts, are revealing new genes [58]. Bioinformatics tools have been developed to correct errors for such platforms [58], allowing reference-free transcriptome analyses [6,59]. This is particularly useful when studying RNA isoforms generated by alternative splicing (AS). Its dysregulation may be responsible for initiation and progression of diseases like cancer. Thus, specific computational tools have been developed to integrate genomics and transcriptomics, for a proper characterization of alternative splicing in health and disease [60], including mitochondrial diseases [34,61]. In relation to that, long-read isoform quantification and analysis (LIQA) allows to identify differential alternative splicing (DAS). Such tools have been applied to study splicing events in cancer [62]. ML approaches, such as deep learning (DL), have been used to analyze the effect of disrupting splicing on pathogenicity [63]. New sequencing technologies also allow novel immunotherapy strategies, to fight cancer and other complex diseases. Interestingly, cancer cells usually exhibit transcriptomics dysregulation. In this scenario, tumor antigens (TA) can be designed from aberrant transcripts encoding cancer-specific proteins. Additionally, big data approaches are used to analyze multi-omics data from cancer cells. Such knowledge allows translating experimental results into new, more efficient therapies with an unprecedented power [64].

Total RNA, poly(A) RNA and non-coding RNA populations can be isolated from tissues or cell cultures. Yet, such approaches can only generate average results, corresponding to such cell populations. Fortunately, it is now possible to isolate RNA from single cells and even from single nuclei. That allows an unprecedented dissection of transcription within millions of individual cells. Both single-cell RNA sequencing (scRNA-seq) and single-nucleus RNA sequencing (snRNA-seq) have exciting applications [65,66,67,68,69,70,71,72,73,74,75,76,77], for instance: (i) discovering and characterizing cell type in health and diseases, such as cancer [13,78,79,80,81,82,83,84,85,86,87,88,89], with implications in immunology [90], immune-mediated diseases [91], immunotherapy [92,93,94,95,96,97,98,99,100] and drug resistance [101]; (ii) deciphering the roles of such specific cell types in health and disease [102], including mitochondrial heteroplasmy [33]; and (iii) analyzing cell emergence, development and plasticity in tissues and organisms. These studies are also applied to study plant biology [103,104]. Currently, sc/snRNA-seq are extensively being used in neuroscience research, including analyses of neurodegenerative disorders at the molecular level. This includes Parkinson’s disease (PD) [105] and Alzheimer’s disease (AD) [106]. Likewise, the development of the human brain from fetal to adult stages has been analyzed at the single-cell level. Interestingly, spatial transcriptomics allows to generate location maps of gene expression within cells, tissues, organs and whole organisms, comparing health and disease [66]. This can be done using probes with single-molecule fluorescence in situ hybridization (smFISH) [107], as well as sequencing with Slide-seq, which has ~10 µm spatial resolution [108,109].

On the other hand, cell identity is determined in different ways, with transcription factor (TF) networks playing an essential role. Recent developments in nucleic-acid sequencing, in general, and sc/snRNA-seq, in particular, allow to couple transcriptomic maps with cell identity, defining profiles of gene expression for each cell [110,111,112,113,114,115]. Interestingly, although pseudogenes were considered functionless, TGS has allowed to identify many transcribed pseudogenes, including protein-coding ones in normal and cancer human cells [116]. Transcriptomics has also been used to study cellular communications, including both intra- and inter-cellular signaling networks [117,118,119,120,121,122]. On the other hand, genome-editing technologies such as CRISPR can be combined with scRNA-seq applied to animal models and human organoids, to shed light on poorly understood diseases like autism [123]. Interestingly, non-coding sequences may be linked to some diseases [32]. As with structural genomics, organelle transcriptomics and mitochondrial disorders are also related to non-coding RNA [37]. Recently, TGS has allowed the sequencing of a class of them known as circular RNA (circRNA), which was previously refractory to sequencing [124].

It should also be taken into account that different sequencing platforms have advantages and disadvantages. Therefore, a combination of several of them may be needed for a comprehensive analysis of gene expression [125]. Besides, computational models [126], such as ML, have been applied to these studies [127], including dimension reduction methods [128]. Bioinformatics developments have also allowed to deconvult heterogeneous cell samples [129], as well as identify pathways or biological processes from transcriptomics [130]. As an example, the worldwide impact of rare diseases is significant, affecting ~350 million people. Nearly 6000 of them have been characterized at the molecular level, but diagnosis remains challenging. Thanks to the new sequencing developments, transcriptomics coupled with ML are being used to diagnose diseases, in general, and rare disorders, in particular [131].

### 2.3. Epigenomics

Epigenetic modifications may change chromosomal architectures, without modifying nucleic acid sequences. Depending on the cell type (prokaryote or eukaryote), different mechanisms may be involved in epigenetics, such as DNA methylation and histone acetylation, modulating different activities. Prokaryotic chromosomes lack histones. Therefore, DNA methylation is a main epigenetic regulator in such cells. There are three types of DNA methylation in prokaryotes: 6-methyladenine (6 mA), 4-methylcytosine (4 mC) and 5-methylcytosine (5 mC), including both bacteria and archaea. New sequencing technologies have allowed to characterize prokaryotic epigenomes [132], with recent developments such as Nick-seq. Thus, datasets are mined to increase sensitivity, specificity and accuracy. This way, genomic maps of DNA modifications and damage are generated, with single-nucleotide resolution [133]. Other new technology allows identification of sulfur replacing nonbridging phosphate oxygen, which is common in prokaryotes, through selective fluorescent labeling of single-stranded DNA phosphorothioate (PT) modifications [134].

The development of TGS capable of reading single molecules has allowed a comprehensive study of frequency and distribution of epigenetic modifications. This way, it has been possible to discover that they may be related to different functions, including regulation of gene expression, maintenance of genome stability, cell cycle, sporulation, cell shape, biofilm formation, motility, siderophore generation, membrane vesicle production, defense (discriminating self from non-self DNA, like the bacteriophages that can be cut by restrictases), lysogenicity, virulence (including pathogen–host interactions and host colonization) and response to the environment [132,135,136,137,138,139]. These studies are important to identify beneficial, harmless, opportunistic and pathogenic-virulent phenotypes related to health and disease [140]. For instance, it has been proposed that epigenetics are involved in the health effects of probiotics [141]. On the other hand, the relevance of DNA methylation in microorganism toxicity has been demonstrated in relation to *Escherichia coli* strains producing Shiga toxin. Indeed, they were responsible for ice cream- and lettuce-associated outbreaks in Belgium and the USA, respectively [142].

Likewise, it has been shown that inactivating 4 mC methyltransferase in *Leptospira* spp. pathogens produced genome-wide dysregulation of gene expression. Epigenetic studies have been also carried out with *Mycobacterium tuberculosis*, which is the infectious agent causing tuberculosis [143]. These findings are particularly relevant in the current trend of antibiotic resistance, with increasing numbers of total drug-resistant (TDR) bacteria resistant to all known antibiotics (known as “super bugs”) [144]. Indeed, TDR *Mycobacterium tuberculosis* strains have arisen in the last two decades, mainly due to the misuse and abuse of antibiotics. This highlights the need for new prevention and treatment strategies for pathogenic bacteria, finding alternatives to antibiotics. The new sequencing technologies are being used to reach such a goal [145]. In this scenario, highly conserved DNA methyltransferases (MTases) are potential targets for epigenetic inhibitors to fight infections [139]. Besides, they may have potential biotechnological applications [146]. Additionally, they represent a valuable tool for aligning metagenomic contigs and scaffolds, preventing errors, as well as assigning mobile genetic elements (MGE), such as transposable elements (TE), to their host genomes [135].

Epigenetics is also important in plants. Being sessile organisms, they have developed regulatory mechanisms to fight abiotic and biotic stresses. This way, approaches such as DeMEter (DME) coupled with quantitative PCR (DME-qPCR) have been developed to quantify DNA methylation in plants. This has been demonstrated in Arabidopsis (*Arabidopsis thaliana*) and tomato (*Solanum lycopersicum*) [147]. On the other hand, 5 mC is involved in regulation of gene expression, repair, replication, transcription, recombination and transposon suppression in plants. The new sequencing platforms have allowed researchers to discover that 6 mA upregulates gene expression, both in eudicots, such as Arabidopsis, as well as monocots, such as rice (*Oryza sativa*) [21]. On the other hand, transposable elements may allow selective advantages and evolution in plants. However, they can also be harmful to their genome integrity, if not properly controlled. The latter can be accomplished through DNA methylation. Thus, it has been found that both 6 mA and 4 mC are involved in TE control of fig tree (*Ficus carica*) [148]. Interestingly, some stress responses are memorized (somatic epigenetic memory), and sometimes they are even inherited through meiosis (transgenerational epigenetic inheritance). This has potential applications to engineer stress-tolerant crops, especially in the current trend of global warming and climate change [149].

Virulence, as well as host and environmental adaptation of different plant pathogens, is also modulated by epigenetics. Examples include fungi and fungi-like microorganisms, such as *Phytophthora* spp. [150]. Interestingly, epigenetics can also be used to protect crops, using sustainable and ecologically-safe biocontrol strategies. For instance, TGS has been used to study biopesticides based on plant growth-promoting rhizobacteria (PGPR) such as *Bacillus velezensis* [151].

Additionally, epigenetics is directly and indirectly related to evolution, enhancing phenotypic plasticity [152], such as thermal adaptation. In this scenario, it is especially relevant for adaptation to present and future environmental conditions [153]. New sequencing methodologies allow to study epigenomics with an unprecedented resolutive power, including reduced-representation bisulfite sequencing (RRBS) and whole-genome bisulfite sequencing (WGBS), analyzing full genomes [154]. This has significant implications in many areas, such as ecology [155], environmental pollution including radiation [156,157], with relevant implications for cancer radioresistance [158] and health [135,138], as well neuropsychiatric disorders [159]. Besides, it has been found that mechanotransduction is involved in mechanical regulation of transcription and the epigenome, having a key role in cancer progression [160]. Interestingly, there is also a link between DNA damage and epigenetics. In this way, it has been found that 8-oxo-7,8-dihydro-2′-deoxyguanosine (8-oxodG) may modulate epigenetic regulation of gene expression [161].

Besides, as with genomics and transcriptomics, mitochondrial diseases have also been linked to organelle epigenetics [37]. Likewise, it is possible to study epigenomes of organisms, tissues, cells and cellular compartments and organelles such as nuclei, mitochondria and chloroplasts. Indeed, whole genome bisulfite sequencing has allowed researchers to demonstrate that methylation patterns are cell type-specific [162]. That opens the door to decipher how genomic regulatory networks work [102]. Interestingly, these findings are particularly relevant for personalized treatments of complex diseases, such as cancer, diabetes and asthma, as well as chronic age-related diseases, due to the interaction of multiple genetic and environmental factors [13,163,164]. Indeed, new sequencing technologies have allowed epigenetic profiling of different cancers [78,165]. It has been proposed that DNA methylation of probiotics plays an important role in immune responses of allergies, autoimmune disorders and cancer. This is mediated by regulatory T cells (Tregs). They are responsible for maintaining tolerance to self-antigens, preventing autoimmune diseases [166]. Treg cells are also subjected to epigenetic regulation. Therefore, an appropriate regulation in such cells, gut microbiota and their interaction is of paramount importance to maintain Treg function, preventing diseases. This is accomplished through transcriptional and epigenetic regulation [167].

On the other hand, developmental trajectories have also been studied. In this manner, it has been possible to identify particular cells responsible for expressing genes related to neurodevelopmental diseases [168], as well as changes during learning and memory [169]. Also, epigenetics have been related to dementia, such as Alzheimer’s disease [170]. Such epigenetic modifications can be quantified not only in the central nervous system (CNS), but also in the cerebrospinal fluid. That opens the door for the development of biomarkers for early detection and treatment of AD [171]. Nevertheless, new bioinformatics developments are still needed, integrating multiplexed assays to better analyze health and disease [19]. An example in such direction is GermLine cycle Expression Analysis and Epigenetics (GLEANE) [172].

Epigenetics can also be applied to study environmental genotoxins causing mutations and cancer. Among them is acrylamide, which can be generated in foodstuff and beverages subjected to high temperatures, as happens with fried potatoes or coffee [173,174]. Acrylamide may generate brain tumors in general and glioblastoma in particular. This is the most aggressive and invasive brain tumor, with a life expectancy between one and one and a half years. Fortunately, new sequencing platforms such as SGS and TGS are significantly increasing our understanding of such diseases. This allows designing molecular markers and analyzing epigenetic profiling at the single-cell level, for better diagnostics, prevention and treatment [20]. On the other hand, recent discoveries have shown that epigenomics, in general, and social epigenomics, in particular, can also be used to ascertain how adverse social factors can generate diseases, especially in childhood [175]. Computational, statistical and bioinformatics tools are also needed to fully analyze epigenetics. In this scenario, as reported for transcriptomics, epigenetics has also been linked to rare diseases using ML, and particularly DL, approaches [176].

### 2.4. Metagenomics

Microbial communities are relevant in different areas, including human and animal medicine, food technology, agronomy, aquaculture and ecology. This way, they have important implications in health and disease, optimizing food and foodstuff production, breeding, biodiversity protection and the fight against the current trend of global warming and climate change. The new sequencing methodologies are opening the door to an unprecedented, powerful study of microbial communities [177,178]. In this way, many new species have been discovered [179,180]. This is contributing to identify healthy microbiomes, as well as diseases linked to dysbiosis scenarios [181]. Altered microbiome profiles have been found in many diseases, not only for typical infections, but also for other disfunctions, such as cancer [165,182]. Nevertheless, results obtained in different experiments may be different, due to experimental biases that must be properly addressed [183]. As with other genomic, transcriptomic and epigenomic areas, microbiome analyses (microbiomics) require appropriate bioinformatics tools [184]. TGS is particularly useful in metagenomic analyses, since it can be used to generate almost or even complete genomes with single reads, significantly reducing or not requiring contig assembly [185]. Therefore, TGS platforms are being used to find microorganisms present in human microbiomes, foodstuff and beverages like milk, aquaculture, soil and many other ecological niches, allowing to identify both beneficial and pathogenic microorganisms [186,187,188], including serotypes with closely related, or even the same, antigenic formulae [189].

Additionally, metagenomics can be used to study biological entities like virusoids, viroids plasmids and viruses [190], including viral quasispecies [191]. For instance, viruses responsible for hepatitis have been identified with short-read sequencing [192]. Long-read sequencing is even better, allowing single reads of full genomes. However, they may require high DNA concentrations, generating more sequencing errors than short-read platforms. Specific workflows combining wet-lab and bioinformatics pipelines have been developed to overcome these limitations. An example of such a strategy is viral metagenomics via MinION sequencing 2 (VirION2). Likewise, bioinformatics tools have been developed to increase long-read quality of sequencing [193]. As expected, short-read sequencing approaches failed to identify biodiversity that was found by long-read platforms, showing significantly higher biodiversity. The methodology has been further optimized to use samples with low nucleic acid concentrations, which may be especially relevant for environmental studies [194].

As with other biological systems, multiple-omics technologies open the door to longitudinal holistic approaches of microbial genomics. Thus, metagenomics, metatranscriptomics, metaproteomics and meta-metabolomics allow to generate an integrated picture of structure, function and phenotype. This opens the door to identify new functions, and even previously unknown species, with a better understanding and prediction of microbe–microbe and microbe–host interactions, with important microbiological, medical, agronomical and biotechnological implications [67,195].

## 3. Future Prospects and Concluding Remarks

The future is certainly promising for nucleic-acid sequencing, mostly due to the ingenious developments of new technologies. One interesting application area of nucleic-acid sequencing is food biotechnology, to identify pathogens. As an example, the IBM DNA Transistor <https://www.ibm.com/ibm/history/ibm100/us/en/icons/dnatransistor> (accessed on 27 July 2021), is being co-developed with Roche to identify pathogens in milk, as well as early detection, prevention, and personalized treatment of diseases. As Gustavo Stolovitzky (Manager of Functional Genomics and Systems Biology Group at IBM) said: “What is the next big thing in biotechnology? The answer is kind of simple if you’re in the field—you need to know how to sequence DNA, fast and cheap”. On the other hand, since TGS allows researchers to directly sequence single molecules, without biases associated with retrotranscription and amplification, that opens new fields of functional genomics. All these breakthroughs, coupled with fewer starting materials required, longer reads and faster turnaround at lower prices, should boost scientific research and discoveries in areas related to living entities. These include medicine, agronomy, ecology and biotechnology.

These developments are relevant, not just for single specimens, but also for population studies, from microbes (metagenomics) to other analyses involving plants and animals. Technological developments and optimizations should generate more detailed and accurate results, allowing researchers to reach new insights and draw more accurate conclusions. In this manner, previously unattainable projects may be possible, for instance, to directly sequence nucleic acids when they are so scarce that FGS and SGS may generate negative results, since TGS can sequence single molecules. Likewise, deciphering what made us human is a provocative topic in biomolecular research, among other exciting research goals, in relation to the new sequencing platforms. In particular, research on non-coding RNA (which typically are short molecules) is particularly exciting, given the surprising implications of spurious or pervasive transcription in organic and cognitive evolution [196,197]. In this way, recent discoveries accomplished by nucleic-acid sequencing are redefining the concepts of gene and transcript.

All these developments, in general, and nucleic-acid sequencing, in particular, coupled with genome-editing breakthroughs, such as CRISPR, are highlighting the relevance of biomolecule analyses and applications. One of the goals is to re-sequence the human genome from the current 1000 USD price to just 100 USD, as shown by the National Human Genome Research Institute (NHGRI): “The Cost of Sequencing a Human Genome” <https://www.genome.gov/about-genomics/fact-sheets/Sequencing-Human-Genome-cost> (accessed on 27 July 2021) [198]. Thus, everyone could have their genome sequenced in the near future. The implications for truly personalized medicine, with much more accurate and efficient diagnosis, prevention and treatment of diseases, will be unprecedented. This includes humans and other animals (veterinary medicine).

Additionally, associating nucleic-acid sequencing to activity-dependent labeling should allow to link transcriptomics and epigenomics with important functional implications, including roles of cells in physiology. New insights will be reached unifying nucleic-acid sequencing with functional, physiological, morphological and phenotypic data. All such research is now generating and will continue to produce huge amounts of data, requiring new software and hardware developments to properly analyze them. This includes AI, ML, DL and neural network chips, such as neural engines. Furthermore, new frameworks will be required to systematically filter, sort and organize such vast knowledge. This should make it easily available in a graphical way, for easier visualization and interpretation. It is clear now that this century will be revolutionary for several scientific areas, including molecular biology and biotechnology related to biomolecule research, with important implications and applications.

## Data Availability

Not applicable.
